# Study on the evolution mechanism of damage to overburden fissures of repetitive mining of close coal seam groups by positive faults

**DOI:** 10.1038/s41598-025-05212-9

**Published:** 2025-06-06

**Authors:** Zuhao Xu, Xiangtao Kang, Dongbin Huang, Chao Ren, Pin Cao

**Affiliations:** College of Mining, Guizhou University, Guiyang, 550025 China

**Keywords:** Proximal coal seam groups, Normal fault, Overburden fissures, Fissure characteristics, Stress evolution, Energy science and technology, Engineering

## Abstract

For the safety of coal mines and the efficient use of gas resources, precise identification of gas transmission routes and enrichment zones is essential. The mining of the coal seam causes many fissures in the overburdened rock, and the evolution of mining fissures is intimately linked to gas transport channels.In order to study the characteristics of overburden fissures in the mining of close coal seam groups under the influence of faults, Shucheng Mining Area in Guizhou as a Research Context, Research using physical similarity simulation tests and numerical simulation methods. The results show that the location of the overburden rupture line interacts with the faults and together they construct a stable inverted triangular overburden stability structure; Given the existence of faults, the upper seam is subjected to greater stress during mining compared to the lower seam, which makes the fissure development process in the upper seam more active.Mining significantly affects the stress distribution of the overlying rock layer of the upper disc coal seam. The stress of the upper disc coal seam increases with the advancement of mining of the lower disc coal seam, and the stress suffered by the side with fewer fault gaps is greater. The results of the study can provide a theoretical basis for mining close coal seam groups in fault tectonic regions, and promote coal mines to achieve safe and efficient use of gas.

##  Introduction

As the food of China’s industrial system and the cornerstone of national economic development, coal plays the dual role of energy and chemical raw materials^1–4^. The stable supply of coal has an irreplaceable strategic position in supporting China’s industrialization process and promoting sustained economic growth. In the coal production areas of Guizhou, close coal seam groups are common, which are characterized by the close distribution of multiple coal seams in the vertical direction, with relatively small spacing between the seams, and all of them have economic mining value. However, the coal mine safety situation is not optimistic, due to the influence of complex geological formations, increasing the difficulty of mining, the mine operating environment has gradually from the original single, easy-to-control mining conditions into a more complex, variable mining conditions stage. The complexity of geological formations is not only reflected in the local regularity of the distribution of rock layers, and the existence of fracture zones and folds, but also may lead to changes in the water table, differences in the mechanical properties of rocks, and an increase in the risk of potential geological hazards^5–12^. In mineral resource extraction activities, the distribution of fault structures poses potential hazards to the structural stability of underground coal and rock seams and the safety of mining operations^13,14^. Therefore, it is of great significance to study the characteristics of overburden fissures in the mining close coal seam group under the influence of faults.

The fault tectonic region is prone to local stress concentration and other phenomena, resulting in mining process working face mining stress distribution abnormality, easy to cause mine power disaster, serious deformation of the roadway, impact pressure, etc., seriously affecting the safety of coal production^15–17^. For this reason, many scholars have carried out research on the characteristics of the mining law under the influence of faults^18^. The asymmetric deformation mechanism exhibited by the rock mass around a near-fault roadway (NFR) under the influence of a mine is revealed using a combination of theoretical analysis, numerical simulation techniques, and field tests^19^.. A kinetic model of rock slip in fracture zones was constructed to characterize the mechanical distribution of rockbursts induced by the interaction of mining activities and faults. The results show that the distribution of shear stress increments is larger in the lower disc of the fault than in the upper disc^20^. The discrete element numerical analysis software 3DEC was used to systematically analyze how parameters such as fault dip, mine size, and fault zone thickness affect the distance between the mining area and the fault, as well as the depth of burial at the upper boundary of the fault^21^. FLAC3D numerical simulation was used to simulate and analyze the evolution of mining stress, fault activation mechanism, and fault energy release characteristics of the lower and upper working faces when they encountered a positive fault during the advancement process. Based on FLAC3 D three-dimensional numerical simulation, Atsushi Sainoki et al.^22^ constructed a geomechanical model of steeply dipping faults with parallel plate ore bodies, and analyzed the influence of fault friction angle, mining depth, distance from ore bodies, and mining rate on the dynamic response of faults. The shear displacement increment and seismic energy release are quantified through static-dynamic analysis, and the evolution law of fracture velocity and slip rate of mining-induced fault slip is revealed. Fault sliding displacement and energy release were found to be significantly enhanced on hanging-wall faults, at low friction angles. At large mining depths, supershear rupture may exacerbate far-field vibration hazards. Taking the D-2 main roadway of the ‘Borynia-Zofiówka-Jastrzbie’ coal mine as the research object, Małkowski Piotr et al.^23^ analyzed the deformation characteristics and support optimization direction of roadway in fault zone through the combination of numerical simulation and field measurement. Andrian Batugin et al.^24^ proposed the’piston effect’hypothesis, simulated the volume change of fracture space caused by the displacement of fault wall by CFD ANSYS software, and analyzed the fluctuation and migration path of gas pressure. It is revealed that the change of fault contact mode is the key inducement of sudden gas release, and the piston effect aggravates the suddenness of gas migration. Although the above scholars have used multiple research methods to investigate the mechanism of fault activity and the impact of faults on mining in the coal mining process, and have come up with many valuable research results. However, the study of mining fissures in close coal seam groups is more limited. The problem of fault activity and its induced ground deformation is more complex in the context of mining a cluster of coal seams nearby. Mining activities can affect the mechanical properties of fault zones and surrounding rock formations, with unpredictable effects on subsequent mining activities^25,26^. Under the complex geological conditions of multi-seam group mining, many scholars at home and abroad have carried out in-depth research^27^. Numerical and physical simulation research methods were used to investigate the dynamic evolution of the stress field induced by mining activities after multiple pressure releases in close multiple coal seams, the nonlinear interaction mechanism of the stress field between coal seams, and the characteristics of its superposition effect^28^. The effects of mining on the structural stability of the overlying rock strata were analyzed by combining physical simulation experiments and digital scattering techniques, and the dynamic characteristics of stratum displacement and gas seepage during the mining process were analyzed^29^. By using Flac 3D three-dimensional numerical simulation software, the dynamic evolution characteristics of the stress and displacement of coal pillar were investigated. However, at present, there are fewer studies on mining close coal seam groups under the influence of faults.

The coal seam endowment in Guizhou is commonly characterized by faults, high gas, and close coal seams. Therefore, to address the problem of mining close coal seam groups under multi-fracture tectonic geological conditions in Guizhou coalfield. In this study, through the physical similarity test, the results and numerical simulation results are verified with each other, and the results are of great significance to the mining of close coal seam group, which provides a reference for the prevention and control of power disaster in the mining process of similar mines.

##  Geological background of the study

**Fig. 1:**
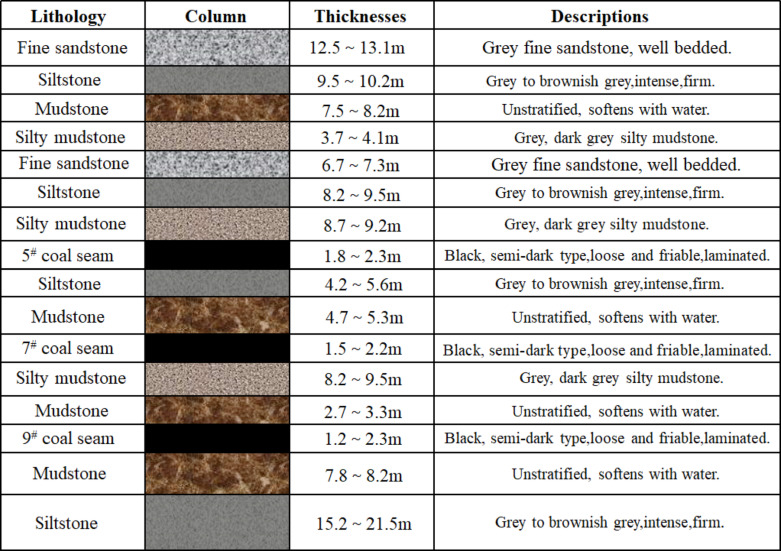
Histogram of coal and rock seams at the working face.

The coal mine stratigraphy belongs to the Permian Upper Longtan Formation stratigraphic system and is located in the Shui Cheng mining area in western Guizhou, China, which is in the transition zone of topographic changes in the central part of the Yunnan-Guizhou Plateau, with unique geological features. Known for its complex and varied geological formations, in which tectonic coals are extremely well developed. Fault structures are widely distributed in the region and are dominated by minor faults. Locally, the distribution of small faults is densely distributed, resulting in a complex coal storage environment, and the recoverable coal seams are often along the backslope axis or flanks. The stratigraphic groups in the Shuicheng mine area are well-developed and clearly defined, with the thin to medium-thickness coal seam groups dominating the area. Coal, mudstone, and sandstone strata were frequently interbedded and deposited in this area, forming a complex stratigraphic sequence with a total stable thickness of between 410 and 420 m. The inclination angle of coal seams is 20° ~ 24°, and the main coal seams mined are 5^#^, 7^#^, and 9^#^, according to the geological exploration report of the Shuicheng mining area in Guizhou in 2022 and the core sampling data of 5^#^, 7^#^, and 9^#^ coal seams, the histogram of mine geological distribution is drawn, the mineColumnar map of geological distribution, is shown in Fig. 1. In the study area, the normal fault structure is mainly developed, and its basic parameters are determined based on on-site geological exploration. The average dip angle of the fault plane is 55°~ 65°, and the maximum drop is about 4.9 m. The fault strength parameters refer to the rock mass mechanics test data of the mining area. The compressive strength of the Longtan Formation of the Permian Longtan Formation is between 26.1 ~ 71.0 MPa, the tensile strength is between 2.03 ~ 9.34 MPa, the elastic modulus is between 22.5 × 10^3^ ~ 37.0 × 10^3^ MPa, the Poisson’s ratio is between 0.19 ~ 0.26, and the water content is between0.06 ~ 2.70%.

## Model similar materials and establishment

### Model similar materials

Based on the similarity theory, the geometric similarity ratio was determined to be 1:100, the volumetric similarity ratio to be 1:1.5, the temporal similarity ratio to be 1:10, and the strength similarity ratio to be 1:150, according to the mechanical parameters of the coal and rock seams. The similarity simulation uses river sand as aggregate and gypsum and lime as cementitious materials, enabling the physical model to maintain overall structural integrity and stability. Mica powder is an auxiliary material for the laminar structure so it forms a lamellar structure, which can construct a laminar structure similar to the actual geological situation and improve the degree of model simulation. Through repeated tests and adjustments, the specific proportions of river sand, gypsum, and lime were determined for the physical and mechanical properties of different coal seams as shown in Table 1, to ensure that the similarity model is highly similar to the actual situation in multiple dimensions, such as geometry, physics, and mechanics. The material of the fault zone is mixed with yellow mud: gypsum: and lime = 6:3:1 to simulate the low strength and layered structure characteristics of the actual fault fracture zone. In the test, the axial stress is applied to the overlying strata by the hydraulic loading system to simulate the actual in-situ stress environment. The vertical stress is set to 0.8 times the prototype in-situ stress. The measured data of in-situ stress in the mining area are referenced, and the step-loading method is adopted to avoid the instantaneous failure of the model.

**Table 1:** Specific parameters for similar material ratios and laying sequences.

No.	Lithology	Proportioning parameters	Thicknesses (cm)	Gross weight (kg)	Grit (kg)	Gypsum (kg)	Lime (kg)
1	Fine sandstone	7:6:4	13	111.69	78.18	20.10	13.4
2	Siltston	8:4:6	10	77.58	62.06	6.20	9.3
3	Mudston	8:2:8	8	71.92	57.54	2.88	11.5
4	Siltstone	8:4:6	4	23.49	18.79	1.88	2.8
5	Fine sandstone	7:6:4	7	60.14	42.10	10.82	7.2
6	Siltstone	8:4:6	5	38.79	31.03	3.10	4.7
7	Siltstone	8:4:6	4	31.03	24.82	2.48	3.7
8	Silty mudstone	8:4:6	5	29.36	23.49	2.35	3.5
9	Mudstone	8:2:8	3	26.97	21.58	1.08	4.3
10	Mudstone	8:2:8	2	17.98	14.38	0.72	2.9
11	5^#^ coal seam	8:5:5	2	10.73	8.58	1.07	1.1
12	Siltstone	8:4:6	5	38.79	31.03	3.10	4.7
13	Mudstone	8:2:8	4	35.96	28.77	1.44	5.8
14	Mudstone	8:2:8	2	17.98	14.38	0.72	2.9
15	7^#^ coal seam	8:5:5	2	10.73	8.58	1.07	1.1
16	Silty mudstone	8:4:6	5	29.36	23.49	2.35	3.5
17	Silty mudstone	8:4:6	4	23.49	18.79	1.88	2.8
18	Mudstone	8:2:8	3	26.97	21.58	1.08	4.3
19	9^#^ coal seam	8:5:5	2	10.73	8.58	1.07	1.1
20	Mudstone	8:2:8	8	71.92	57.54	2.88	11.5
21	Siltstone	8:4:6	22	155.15	124.12	12.41	18.6

### Similarity modelling

**Fig. 2:**
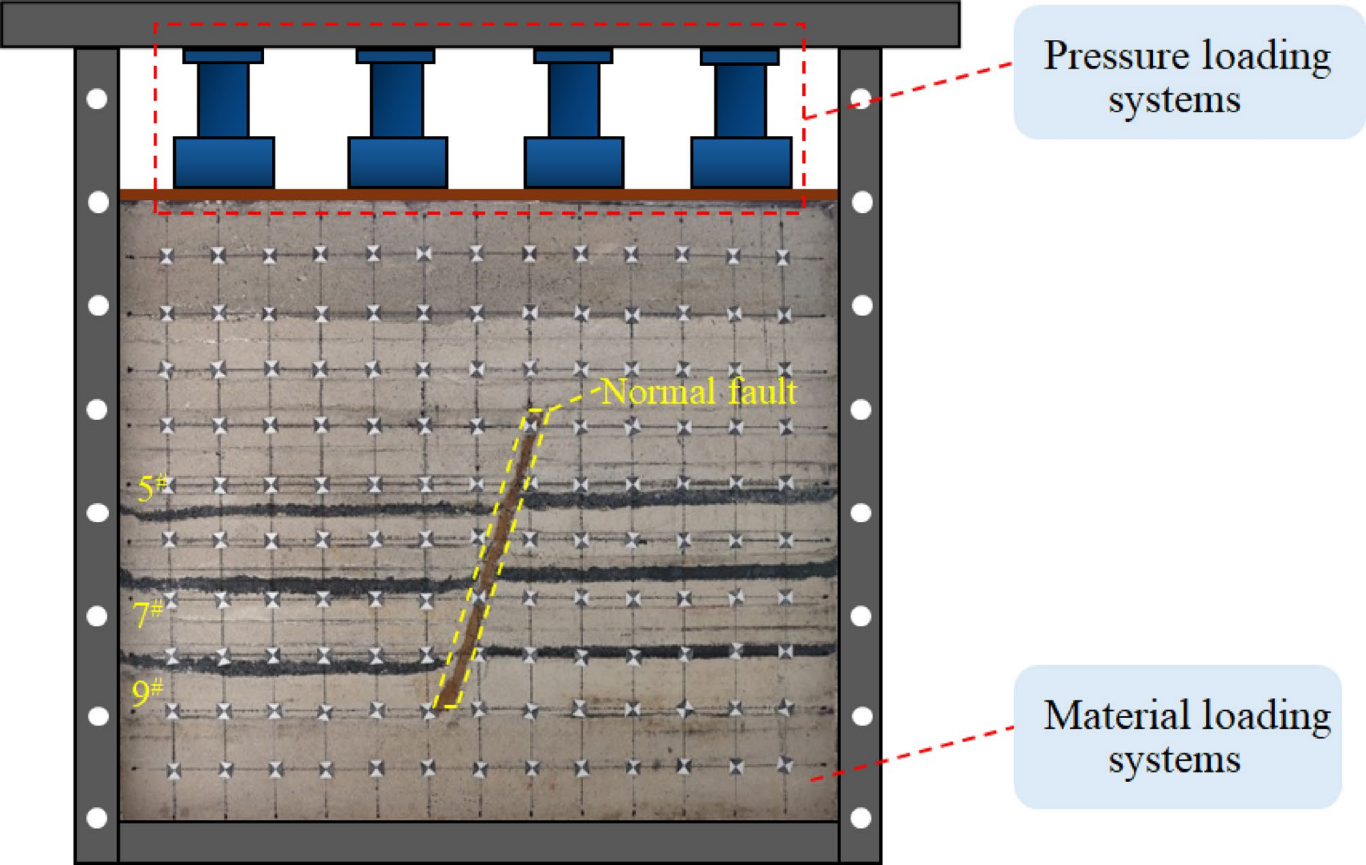
Similar model test charts.

Similar simulation experiments were conducted to investigate the overburden fissure evolution pattern of the mined proximal coal seam group under the influence of simulated faults, ignoring some of the secondary factors that influence the overburden damage evolution. In order to construct a close to the real mining environment, the test adopts the movable multifunctional coal rock layer similarity simulation experiment table developed by our team, as shown in Fig. 2. The dimensions of the experimental model are 140 cm × 40 cm × 160 cm. After a similar model has been laid, two key stages are required to ensure that its physico-mechanical strength accurately reflects the properties of the actual geological body. The first is the natural stabilization period(7 days), a process that promotes the initial integration and solidification of the material between particles. This is followed by a natural air-drying period (14 days), in which the moisture inside the model is gradually dispersed through evaporation, leading to closer contact between the material particles and full curing of the binder, resulting in the densification of the model’s microstructure and a significant increase in its macroscopic mechanical properties.

## Study on the influence of fault structures on mining close coal seam groups

### Regional modeling of fracture characteristics of mining overburden

**Fig. 3:**
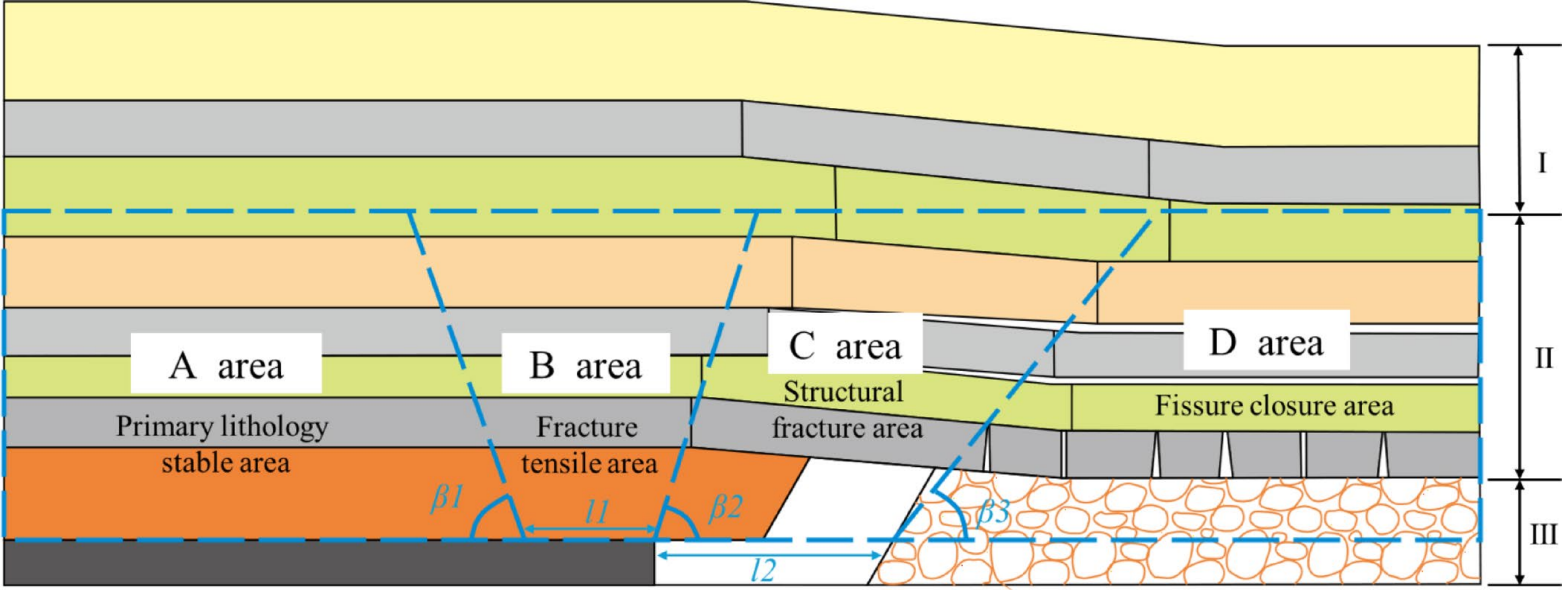
Regional modeling of overburden fracture properties.

In the advancing direction of the working face, the overlying rock layers can be divided into four zones according to their mechanical properties and destructive structural features, from left to right: the primary lithological stability zone (Zone A), which maintains the original nature of the rock and the state of not being significantly disturbed; The fissure stretching zone (Zone B), due to the rock formation is affected by mining so that the original rock stress of the rock formation is changed, the rock formation in the coal pillar in the coal pillar support effect, the cantilever bending characteristics; Structural fracture zone (Zone C), with the advancement of the working face, the overlying rock strata fracture along a certain angle, the rock strata in the broken state, the formation of“masonry beam”bearing structure upward transmission; Fissure closure zone (Zone D), in which the central part of the open area is gradually closed as the overlying rock layers come under pressure periodically, as shown in Fig. 3^30–36^.

In the vertical direction, the overlying rock formations are usually divided into different zones based on the characteristics of fracturing and deformation of the rock formations, in order from top to bottom: Bending and subsidence zone (Zone I), although the rock body in this region has not experienced a direct fracture process, under the joint influence of long-term gravity and the pressure of the overlying rock layer, it gradually shows the overall bending deformation and progressive subsidence characteristics, and the changes in this region reflect the slow adaptation of the rock body to external loads in a long time scale; Fissure zone (Zone II), the rock body in this area is subject to a variety of stresses, the fissure network is highly developed, forming an intricate fissure system, these fissures are not only the channel for stress release but also important pathways for gas transport, which has a significant impact on the permeability and strength of the rock body; Rock Collapse Zone (Zone III), which directly reflects the phenomenon of intense fragmentation and collapse of the rock body under strong stress, is a significant loss of the structural stability of the rock body, which is characterized by the accumulation and displacement of broken materials^37–43^.

### Fracture mechanics of mining overburden under the influence of faults

#### Mechanical analysis of the mining mechanics of the upper disc working face

**Fig. 4:**
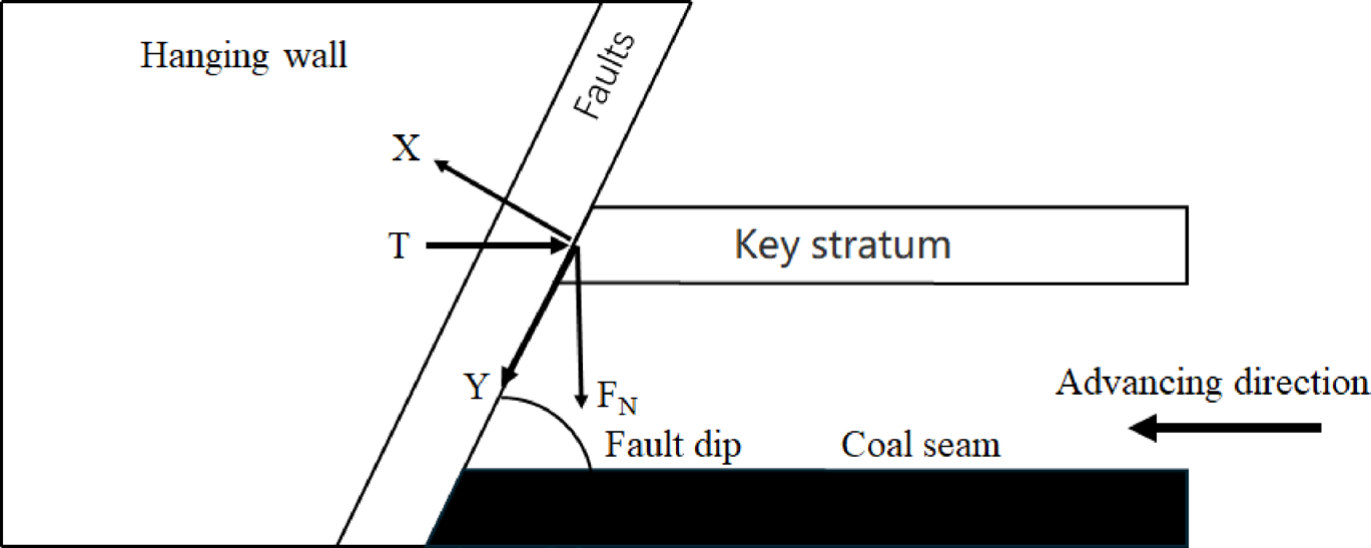
Simplified diagram of mining mechanics of the upper disc working face.

In general, for the simplicity of the analysis process, the fault can be considered as a structurally weak surface. Based on the theory of “Key stratum”, the mechanical analysis structure of the upper disc working face mining is simplified as shown in the Fig. 4. The use of faults as a reference level of study allows for the inclusion of T, and F_N_. The force along the fault plane in the x and y directions is analyzed as^44^:

1 $$\left\{ {\begin{array}{*{20}c} {F_{X} = F_{N} cos\theta + Tsin\theta } \\ {F_{y} = F_{N} sin\theta - Tcos\theta } \\ \end{array} } \right.$$

Equation F_X_, F_y_,is the force in the x and y directions with the fault plane as the reference. K_N_; F_N_, is the support of the fault plane against the key layer. KN; T is the squeezing force of the fault face on the critical layer, and K_N_;θ is the dip of the fault, (°).

It can be seen from Eq. F_X_ is constantly greater than 0, which indicates that the slip of the fault plane and the key layer depends on the magnitude of the squeezing pressure T, and whether or not the two 0 are delaminated has nothing to do with mining.

The mechanical conditions for the keystone block not to slip at the fault are:

2 $$\left| {F_{y} } \right| \le F_{x} tan\theta .$$

Equation θ is the internal friction angle of the fault, (°).

On the spot F_y_ >0 when F_N_ sinθ-Tcosθ > 0 which leads to

3 $$F_{N} /T \ge cot\theta.$$

Bringing Eq. (2) into Eq. (1) yields:

4 $$Tcos\left( {\theta - \vartheta } \right) \ge F_{N} {\text{sin}}\left( {\theta - \vartheta } \right).$$

According to the relationship between the magnitude of the fault angle and the internal friction angle of the fault zone, Eq. (4) can be expressed as:

5 $$\left\{ {\begin{array}{*{20}c} {F_{N} /T \ge \cot \left( {\theta - \vartheta } \right) \theta < \vartheta } \\ {F_{N} /T \le \cot \left( {\theta - \vartheta } \right) \theta> \vartheta } \\ \end{array} } \right.$$

In the structural fracture zone (zone C), the ‘masonry beam’ structure formed by the fracture of the key layer interacts with the fault zone, and its mechanical equilibrium conditions can be described by formula (1)~(5).When the keystone block slips at the fault, the concentration of tensile stress in the fissure stretching zone (Zone B) will exacerbate the extension of the overlying rock fracture line to Zone A, forming the mechanical basis for an inverted triangular stable structure.

Similarly, when F_y_ <0 When Eq. (2) is brought into Eq. (1) to get:

6 $$Tcos\left( {\theta + \vartheta } \right) \le F_{N} {\text{sin}}\left( {\theta + \vartheta } \right).$$

And according to related studies, it is known that the internal friction angle of the fault is generally 38° to 45°, so the sin(θ-ϑ) > 0 is constant, then (6) can be expressed as:

7 $$F_{N} /T \ge \cot \left( {\theta + \vartheta } \right).$$

The mechanical conditions for the fault not to be “activated” during the advance of the upper face can be expressed by the coupling (3), (5) and (7) as follows.

On the spot F_y_ >0 when,

$$\cot \theta \le F_{N} /T \le \cot \left( {\theta - \vartheta } \right)$$

8 $$\left\{ {\begin{array}{*{20}l} {cot\theta < F_{N} /T~~\theta < \vartheta } \hfill \\ {\cot \left( {\theta - \vartheta } \right)\left\langle {F_{N} /T~~\theta } \right\rangle \vartheta } \hfill \\ \end{array} } \right.$$

On the spot F_y_ <0 when,

$$\left\{ {\begin{array}{*{20}l} {cot\theta \le F_{N} /T \le cot\theta } \hfill \\ {F_{N} /T> 0} \hfill \\ \end{array} } \right.$$

#### Mechanical analysis of the mining mechanics of the lower disc working face

**Fig. 5:**
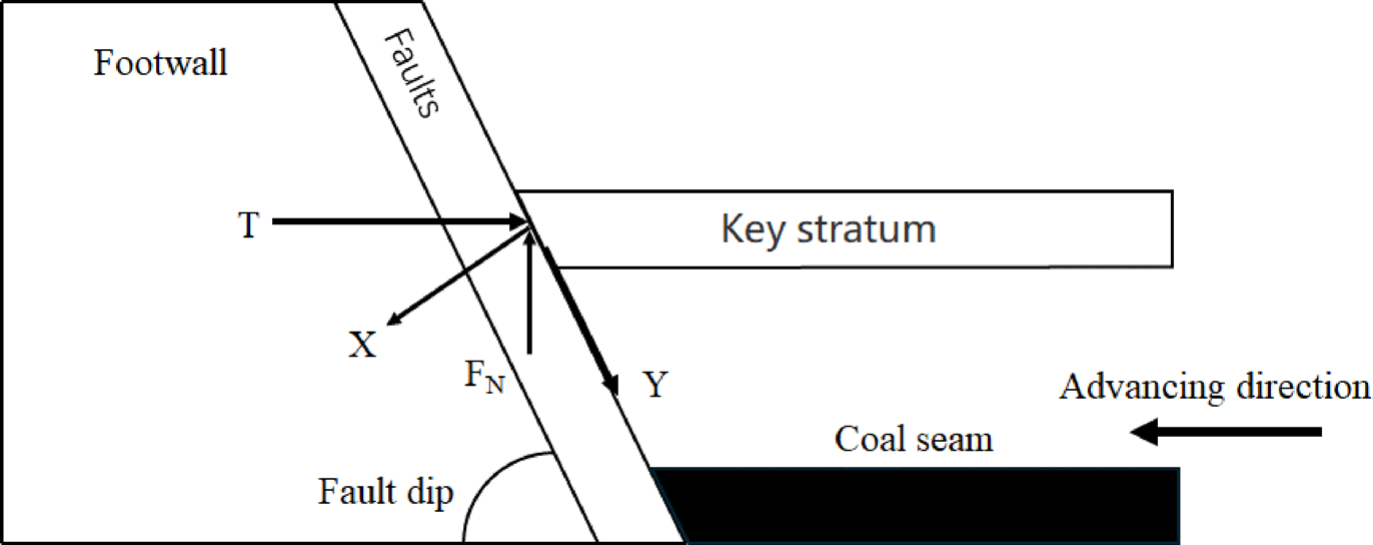
Simplified diagram of mining mechanics of the upper disc working face.

Similarly, a simplified representation of the mechanical analysis of the lower disc workings advancing along the fault, as shown in Fig. 5 while the T, F_N_ The force along the fault plane in the x and y directions is analyzed as:

9 $$\left\{ {\begin{array}{*{20}c} {F_{X} = F_{N} cos\theta + Tsin\theta } \\ {F_{y} = F_{N} sin\theta - Tcos\theta } \\ \end{array} } \right.$$

In order not to cause the key layer and the fault surface to slip away from the layer due to mining activities, the weight pressure of the key layer and the overlying rock layer is transferred to the front coal pillar, and the compression of the stress concentration in the front coal pillar leads to the “activation” of the fault, the conditions must be met first:

10 $$F_{x}> 0 \Rightarrow Tsin\theta - F_{N} cos\theta> 0 \Rightarrow F_{N} /T < tan\theta.$$

At the same time the key layer of rock block at the fault does not slip also to meet the mechanical conditions of Eq. (2), that is, Eq. (2) into the Eq. (9), can be obtained:

11 $$- Tcos\left( {\theta + \vartheta } \right) \ge F_{N} \sin \left( {\theta + \vartheta } \right) \Rightarrow F_{N} /T \le - {\text{cot}}\left( {\theta + \vartheta } \right).$$

The joint Eq. (10) and Eq. (11) satisfy both F_N_, T ≥ 0 That is to say, when the lower face advances, the key layer of rock blocks and faults to maintain relative stability, faults do not occur “activation” mechanical conditions.

When the working face of the footwall is advancing, the stress shielding effect of the primary lithology stable area (area A) can be quantified by formula (9)~(11), which together with the compaction effect of the fracture closure area (area D) inhibits the fault activation, and the shear fracture development degree of the fracture zone (area II) directly affects the stress transfer path of the key layer.

### Characterisation of fissures in mining overburden under the influence of faults

During the mining process of the proximity coal seam cluster, the coal seam mining activities cause significant disturbance and damage effects on the overlying rock structure, and the evolution of the fracture network within the coal rock body is extremely complex. During the mining process, the stress balance of the overlying rock strata in the extraction zone will be affected by the mining activities, resulting in a large number of fissures.

**Fig. 6:**
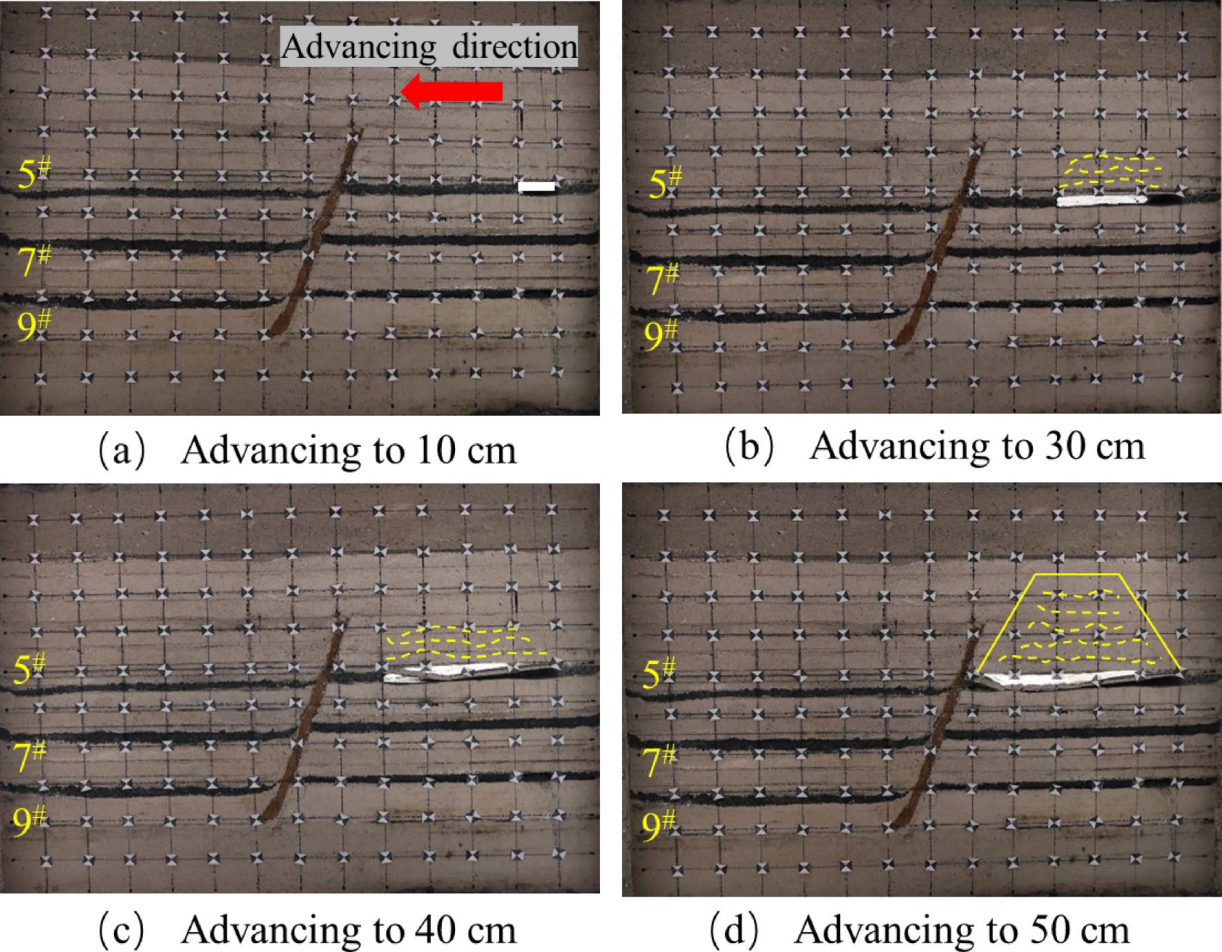
Schematic diagram of the lower disc of the 5^#^ seam.

The tests were carried out using hydraulic jacks to apply the loads and physically similar modeling of the excavation according to the strike longwall collapsed coal mining method. In order to weaken the influence of the boundary effect of the similar simulation test, according to the coal mining conditions, the boundary coal pillar is set at 10 cm from the model boundary at the cutting eye, and the same layer of coal but the fault is mined firstly from the lower disc seam and then from the upper disc seam. When the working face in the lower disc of the 5^#^ coal seam advances to 10 cm, the exposed area of the direct roof is relatively small, and there is no obvious change in the overlying rock layer of the mining area, there are no obvious cracks in the immediate roof, and only a small amount of tensile cracks are produced due to stress adjustment, as shown in Fig. 6a. When it is pushed to 30 cm, the separation cracks begin to appear in the overlying strata, which are mainly tensile cracks distributed at the edge of the goaf. At this time, the number of cracks is small and the density is low, which mainly expands in the horizontal direction. The positioning piece of the direct roof of the footwall working face of the 5 # coal seam produces a slight slip, as shown in Fig. 6b. When the working face is excavated to 40 cm, the immediate roof begins to collapse initially, forming vertical cracks. The type of cracks gradually transitions from single tension to tension-shear composite, and the number of cracks increases. There is a ‘trapezoidal’ distribution of the crack network above the goaf, but the overall density is still low, as shown in Fig. 6c. When pushed to 50 cm, the direct roof further collapsed and the angle of fragmentation increased, and the fissures generated by the mining impact on the overlying rock layer in the hollow zone were distributed in a “trapezoidal” shape. The tensile cracks are mainly distributed on the edge of the goaf and the immediate roof area. The tensile stress concentration of the overlying rock is caused by the exposure, and the vertical cracks are formed. Shear cracks begin to appear near the fault, and the number and density of cracks increase with the expansion of the mining influence range, especially in the edge of goaf and the footwall area of the fault, as shown in Fig. 6d.

**Fig. 7:**
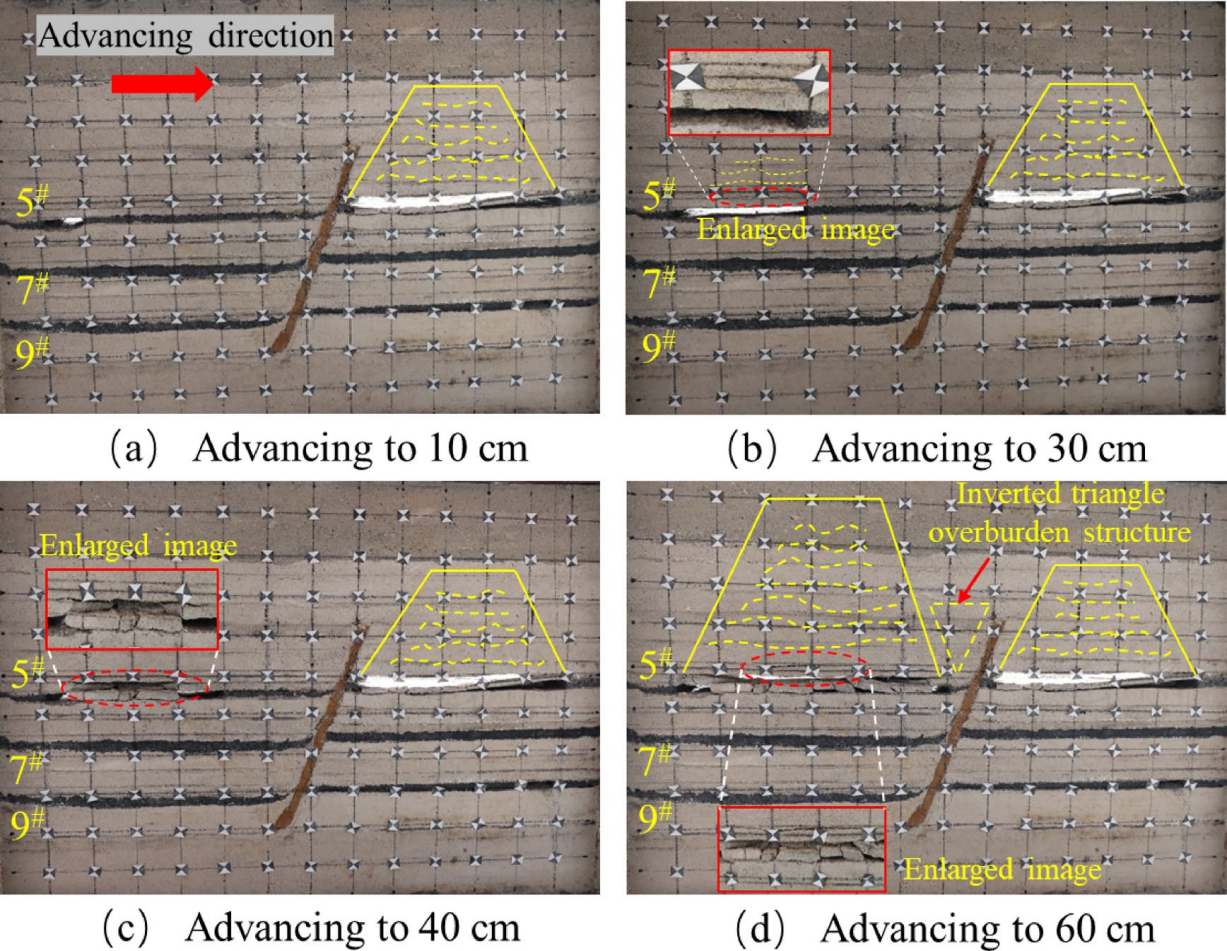
Schematic diagram of the upper disc of the 5^#^ seam.

After mining the lower face of the 5^#^ seam, the upper seam was mined. When the upper disc working face of the 5^#^ coal seam advanced to 10 cm, the upper disc coal seam working face was less affected by mining at this time, and the top plate did not produce obvious fissures, the development of roof cracks is lagging behind, and only micro-tensile cracks appear locally, as shown in Fig. 7a. When the working face advanced to 30 cm, as shown in Fig. 7b, obvious cracks appeared in the overlying rock layer, but there was no obvious layering phenomenon, the fracture type is mainly tensile, but the number is more than that of the footwall at the same mining stage, and oblique shear fractures begin to appear near the hanging wall of the fault, slight slip occurred in the positioning piece of the overlying rock on the upper disc. As the working face pushed forward, due to the limited bearing capacity of the roof slab, when it reached 40 cm, the roof slab broke directly from the middle, and the length of the collapsed roof slab was 21.6 cm. The roof slab near the end of coal mining formed a “cantilever beam” structure. At this time, the tensile cracks and shear cracks are intertwined, the number of cracks increases significantly, and the density is higher than that of the same stage in the footwall, especially in the area near the fault, as shown in Fig. 7c. When advancing the working face to 60 cm, as shown in Fig. 7d, the roof slab collapsed directly, and the development of the overlying rock layer’s detachment fissure was obvious, the tensile and shear composite cracks increased significantly, and the collapse was classically distributed in the form of “trapezoid”. In the process of coal seam advancing, no obvious dislocation of the fault plane is observed in the physical similarity model, but the inverted triangular stable structure is formed in the near-fault overburden, indicating that the fault is in the’critical activation’state. Its bottom corresponds to the’masonry beam’support system of the structural fracture zone (zone C), and the top extends to the bending subsidence zone (zone I), indicating that the interaction between the fault and the overlying rock fracture line has penetrated the fracture zone of zone II and the caving zone of zone III, forming a stress transfer path across multiple regions.

By comparing Figs. 6 and 7, it can be seen that for the same coal seam, the performance of the upper disc working face and the lower disc working face in the mining operation shows a certain degree of similarity. However, when analyzing in detail the geological effects of the extractive activities in both, it is clear that more fissures have been created in the area of the upper workings than in the area of the lower workings. The development of overburden fissures at different advancing distances can be observed from the similar model, and the “trapezoidal” geometries induced by mining operations on the upper face of the 5^#^ seam show a more significant tendency to increase in the vertical direction, compared with the mining process on the lower face of the 5^#^ seam. The “trapezoidal” geometry shows a more significant increase in height in the vertical direction. In addition, the upper plate mining resulted in a significant change in the fracture characteristics, with not only a significantly longer extension of the fracture length but also a significant increase in the number of fractures compared to the lower plate mining. When the upper face is excavated to the vicinity of the fault, the location of the fracture line of the mining overburden and the fault forms a stable inverted triangular structure, as shown in Fig. 7d, the triangle, as the most stable shape structure, is conducive to the stability of the fault, and is not easy to occur “activation”. At the same time, when the working face advances to the vicinity of the fault, the fracture direction of the overlying rock layer is opposite to the dip angle of the fault, and the “trapezoidal” structure formed after the collapse of the overlying rock has a supporting effect on the inverted triangular overlying rock structure. Therefore, the faults are not easy to be “activated”.The excavation of the 5# coal seam has just been completed, at this time, the mining overburden has not yet reached complete stability, the overburden rock layer continues to undergo minor subsidence, and some of the fissures continue to be compacted until they stabilize.

**Fig. 8:**
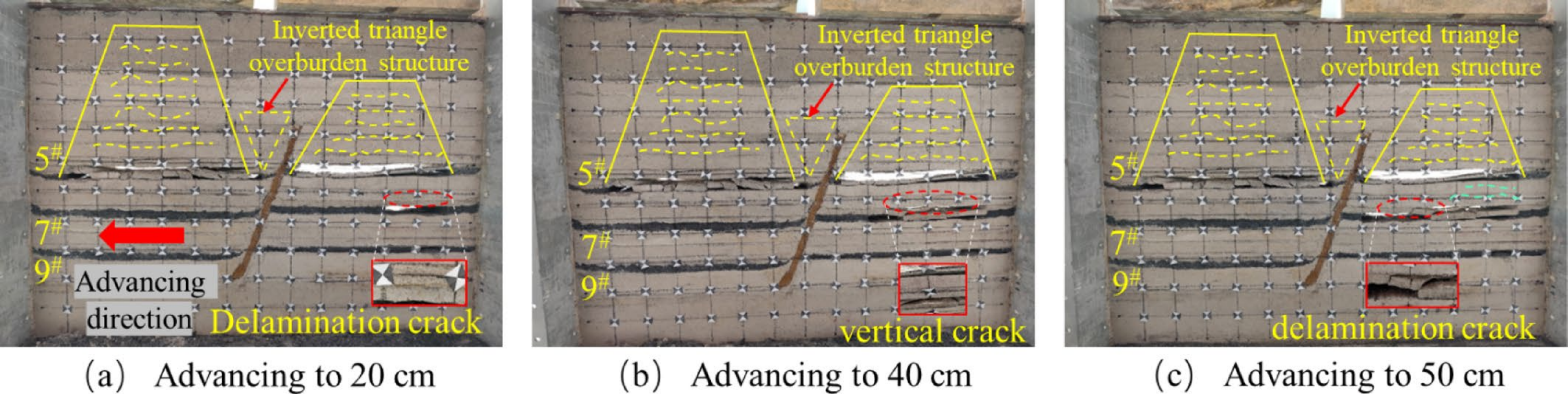
Schematic diagram of the lower disc of the 7^#^ seam.

After mining the 5^#^ coal seam, when the mining of the 7^#^ coal seam continued to advance to 20 cm, there are a small number of bed separation cracks in the overlying strata, mainly tensile cracks, with a small number and scattered distribution. The mining direction of the overlying strata produces a bed separation crack of about 4.3 cm in length, and the positioning sheet of the overlying strata slips slightly, as shown in Fig. 8a. When the working face advanced to 40 cm, the initial rupture of the direct roof occurred, and the rock layer above the direct roof was affected by the stress disturbance from the fracture of the roof plate below, and the local area began to show signs of slight subsidence, and formed a stable “masonry beam” structure, and the local area showed a tendency to separate the layers, Shear cracks caused by stress concentration began to appear in the footwall area of the fault. The number of cracks gradually increased with the expansion of the mining range, and the density increased, as shown in Fig. 8b. When the working face advanced to 50 cm, the roof slab did not collapse further due to the “hinge” structure formed by the rupture of the roof slab at a certain angle, but showed slight signs of subsidence at the 40 cm positioning line, forming a stable “masonry beam” structure, as shown in Fig. 8c.

**Fig. 9:**
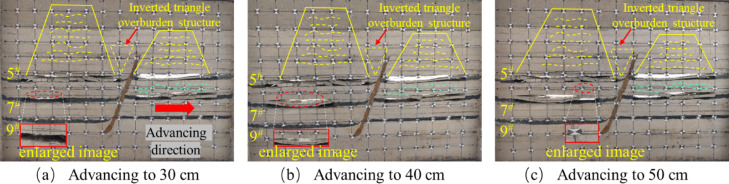
Schematic diagram of the upper disc of the 7^#^ seam.

The excavation of the upper face of the 7^#^ seam was similar to that of the lower face of the 7^#^ seam. The upper face of the 7^#^ seam was advanced until it reached 30 cm, and the overlying rock strata produced an obvious off-stratum fissure, the fracture type is mainly tensile, with a length of about 26.7 cm, and the rock strata above the direct roof did not produce a fissure, as shown in Fig. 9a. After advancing to 40 cm, the roof plate reached its ultimate bearing capacity and then collapsed, and the fissures in the overlying rock layer of the roof plate continued to expand upwards, tensile cracks and shear cracks coexist, and the number of cracks increases significantly, as shown in Fig. 9b. When advancing to 50 cm, the overlying rock layer did not continue to produce horizontal devolatilization fissures because the top slab had collapsed and the top slab collapsed to form a more stable structure, but upward vertical fissures were produced in the overlying rock layer as shown in Fig. 9c. Affected by the mining of the footwall coal seam, when the hanging wall working face advances to the vicinity of the fault, the shear fracture near the fault plane can be seen in the physical model to expand radially, but no penetrating slip zone is formed.

The upper face of the 7^#^ seam was first mined at a greater distance than the lower face for the first time to produce off-seam fissures, and the length of the fissures produced was much greater than that of the lower face, for the following reasons: Firstly, the fault geological structure changes the stress distribution of the coal seam, so that the upper disc working face and the lower disc working face are not subject to the same stress. Secondly, according to the actual production situation of the coal mine, the same coal seam but the fault first digging the lower disc working face, so that the upper disc working face will be affected by the mining of the lower disc working face. Finally, the angle and length of the initial collapse of the roof slab at the upper working face of the 7^#^ seam is greater than that at the lower working face. As the upper working face is subjected to greater stress than the lower working face under positive fault geological structure conditions^45^, the greater stress will lead to easier expansion and penetration of cracks and weak surfaces within the roof slab and overlying rock layers, thus increasing the angle and length of the collapse. When the upper face of the 7^#^ seam was mined near the fault, it was affected by the fault and the mining movement of the lower face, and the overburden fissures above the face were highly developed both vertically and horizontally. In contrast, when the lower workings were mined near the fault, only horizontally developed off-grade fissures were produced.

**Fig. 10:**
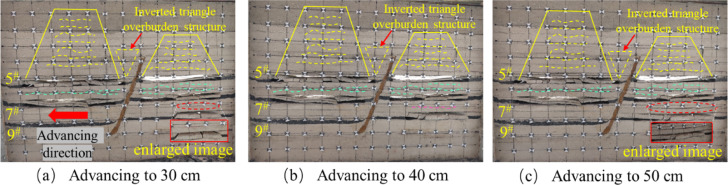
Schematic diagram of the lower disc mining of 9^#^ coal seam.

The overlying strata of the footwall working face of the 9^#^ coal seam did not change during the excavation process. When it was pushed to 30 cm, the roof collapsed immediately, the rock caving zone was compacted, and the overlying strata produced separation cracks, mainly tensile cracks, with a small number concentrated in the middle of the goaf, as shown in Fig. 10a. When advancing to 40 cm, the overlying strata collapse at a certain angle to form a ‘hinge’ structure with the compacted roof of the rock caving zone. The tensile cracks expand along the horizontal direction, and the shear cracks appear less in the footwall area of the fault. The number and density of cracks are slightly lower than those of the footwall mining of 7^#^ coal seam, as shown in Fig. 10b. When advancing to 50 cm, the collapse angle of the roof plate increased, forming a new “hinge” equilibrium. The upper overburden rock bent and sank to form a “masonry beam” structure, resulting in overburden off-layer fissures, with a fissure development length of 49.4 cm, the development of shear fractures near the fault is limited, and the overall fracture characteristics are mainly tensile, as shown in Fig. 10c. When the hanging wall working face is mined near the fault, the tensile cracks in the fracture tensile zone (zone B) and the shear cracks in the structural fracture zone (zone C) are interconnected, resulting in a significantly higher fracture density in the fracture zone of zone II than that in the footwall.

**Fig. 11:**
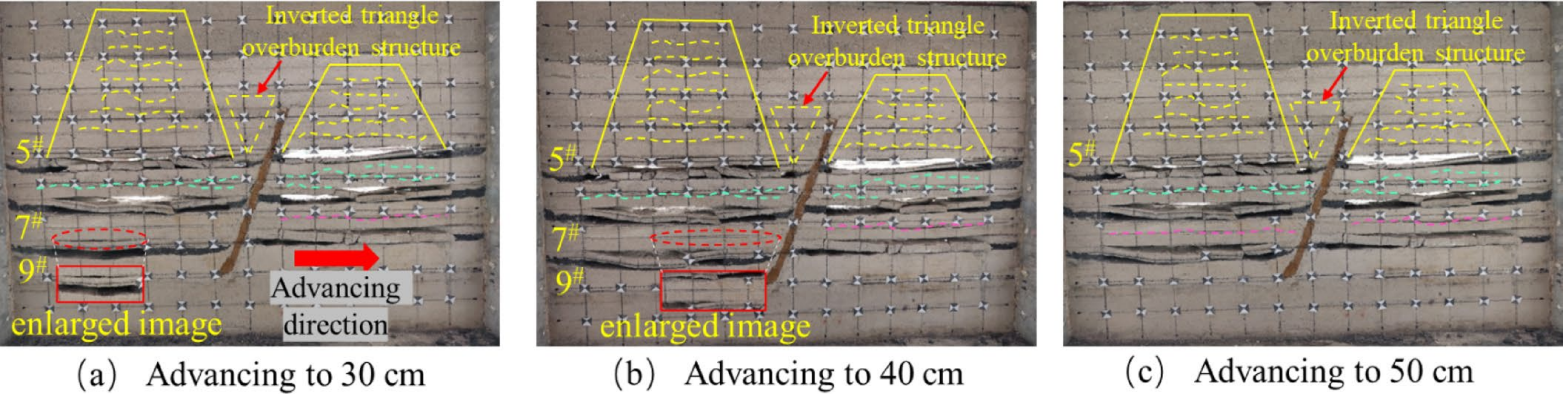
Schematic diagram of the 9^#^ coal seam upper disc mining.

When mining the lower face of the 9^#^ seam, the overlying rock layer remained stable and nosignificant changes were observed. When digging to 30 cm, the roof slab bent and sank to form a “masonry beam” structure. The overlying rock layer produces a distinctly discrete fissure, which develops to a length of 28.4 cm, as shown in Fig. 11a. When pushed to 40 cm, the top plate reached the maximum bearing capacity, the top plate fractured and collapsed, the locating piece on the direct top sank 3.6 cm, and the overlying rock above the top plate continued to produce new fissures, as shown in Fig. 11b. When advancing to 50 cm, the angle of roof collapse increases, and the fissures above the collapsed overburden are easy to penetrate the upper coal seam, increasing the scope of overburden fissure network development, as shown in Fig. 11c.

When the 9^#^ seam was first advanced to the same length, different phenomena were produced in the upper disc working face and the lower disc working face. The initial collapse of the lower working face has a higher degree of direct roof crushing, while the upper working face produces larger off-seam fissures. The presence of faults leads to an imbalance in the stress distribution between the upper and lower working faces during the mining process, resulting in an obvious asymmetry in the degree of stress concentration and stress release pattern between the upper and lower areas. The angle of direct roof rupture in the upper workings of the 9^#^ #seam is greater than that in the lower workings. During the mining process, the overlying rock layers on the direct roof of the upper disc face are susceptible to the stretching effect of fault activity, which results in a higher level of stress in the overlying rock of the upper disc face, thus rupturing the direct roof at a greater angle.

#### Numerical modelling studies

**Fig. 12:**
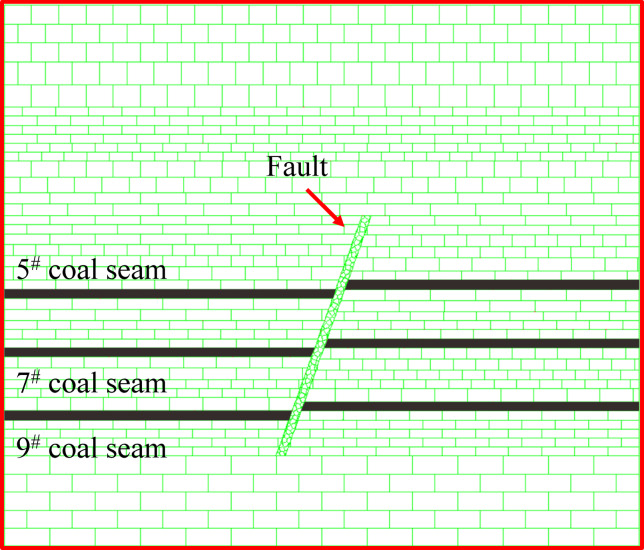
Numerical modelling coal seam and fault distribution schematics.

A numerical computational model was developed using UDEC and the design numerical model dimensions were 140 cm × 120 cm (length × width). The Mohr-Coulomb elastoplastic model was used for the ontological model of the coal rock body, and the face contact Coulomb slip model was used for each block of the joint sur-null. The lower boundary of the model is set as a displacement boundary limiting vertical displacement, the left and right boundaries are set as boundaries limiting horizontal displacement, and the upper boundary is set as a free boundary. At the same time, a stress load was applied to the overlying strata, and the load arrangement was set to a uniform load with a gravitational acceleration of 9.8 m/s^2^. The 5^#^, 7^#^, and 9^#^ coal seams are all near-horizontal coal seams, and the inclination angle of the coal seams are all set to 0° in order to facilitate the calculation. In order to eliminate boundary effects, 10 cm protective coal pillars were installed on the left and right sides of the model. The model scheme is shown in Fig. 12. According to the actual situation of the site, the coal seam is mined in the direction of the fault, but the fault is first mined in the lower disc working face, with 10 cm of excavation each time.

During coal seam mining operations, the internal stress state of the overlying rock strata is redistributed as a result of mining-induced disturbances. When this stress state exceeds the maximum stress threshold that the overlying rock formation can withstand, fracture damage will occur in the rock formation, which in turn will generate mining fissures. The fracture evolution process of the overburden can be divided into three successive stages: generation, expansion and penetration, and compaction and stabilization, and the development rate of the fracture in the overburden and the characteristics of the dislocation of the overburden show variability for different mining heights. Through the UDEC numerical simulation software combined with the actual mining situation of the coal mine, the fissure development map in the mining process of 5^#^, 7^#^, and 9^#^ coal seams were simulated, and the cloud map of the stress distribution change in the excavation process was drawn by using Tecplot, as shown in Fig. 12. The figure shows the development of cracks and changes in stress distribution for different excavation progresses of the model, with positive stress values indicating tensile stresses and negative values indicating compressive stresses. To validate the similarity simulation test, the sequence and length of coal seams were kept consistent with the similarity simulation.

**Fig. 13:**
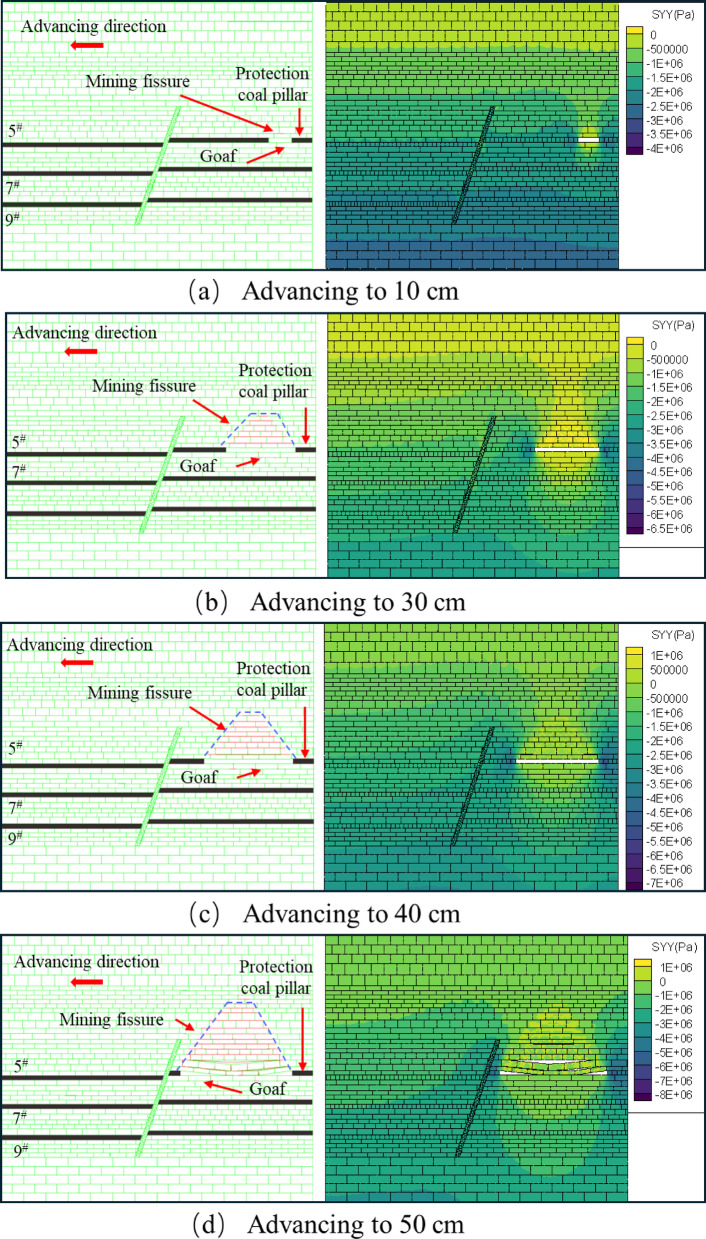
Map of excavated fissure development and stress change in the lower disc of 5^#^ coal seam.

A small amount of fissures were created in the overburdened rock directly above the top after mining began in the lower face of the 5^#^ seam. The two sides of the extraction zone are affected by the supporting stress to form a stress increase zone, the top plate of the extraction zone is affected by mining to form a stress decrease zone, and the overlying rock layer is unloaded, thus generating fissures. As the mining distance increases, the overburden above develops into a “trapezoidal” structure, and the height of the fissure develops to 86.5 cm after the end of mining, with a small number of fissures at the top of the fault and at the upper working face close to the fault. With the increase of the exposed area of the roof plate, the higher the supporting stresses at the opening eye and the working face, and the greater the influence of the roof plate of the mining hollow area by its own weight. As the working face continues to advance, the load borne by the overlying rock decreases, and the decompression zone decompression effect is obvious. When the excavation of the lower face of the 5^#^ coal seam is finished, the left side of the coal seam protects the coal pillar and the fault locally plays the main bearing role, and the stress in this area increases significantly. At this time, the overlying rock layers form a “trapezoidal” structure of the distribution of the pressure relief zone, as shown in Fig. 13.

**Fig. 14:**
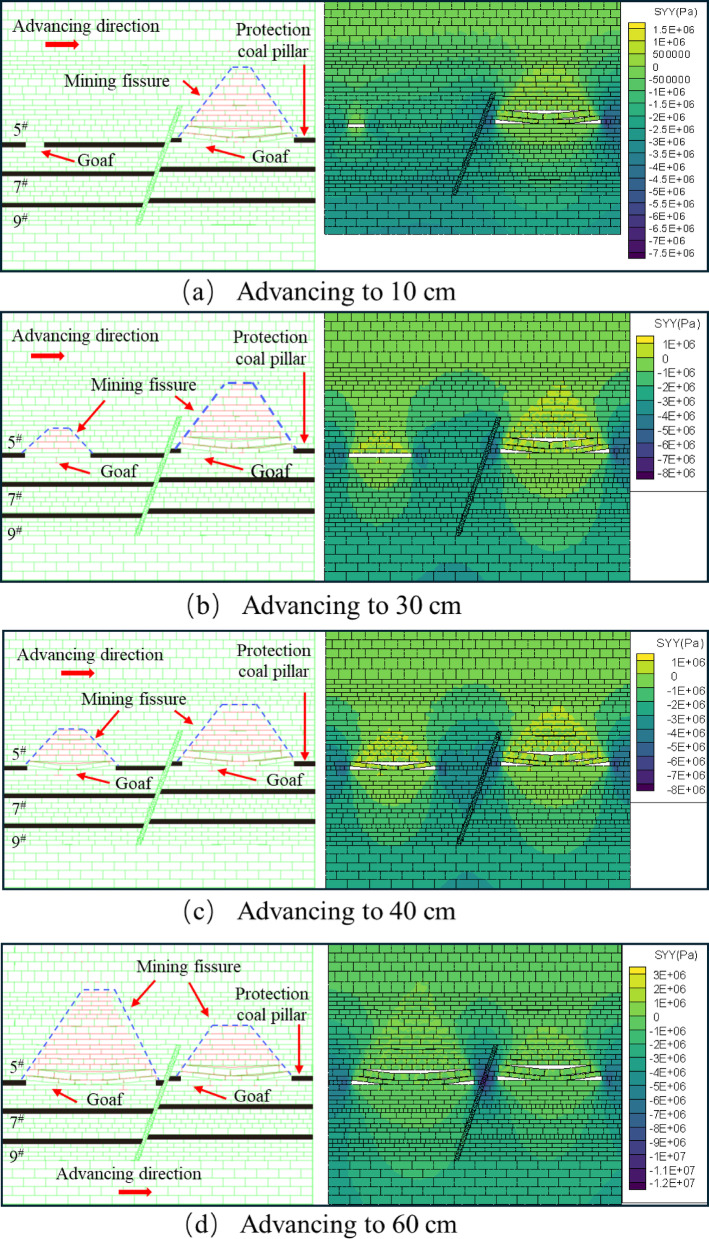
Map of excavated fissure development and stress change in the upper disc of 5^#^ coal seam.

After mining began in the upper disc face of the 5^#^ seam, it did not create a fissure in the overlying rock above, as shown in Fig. 14a. Until advancing to 30 cm, the fissures in the overburden rock above developed into a “trapezoidal” structure, while a small number of fissures were generated in the bottom rock layer, as shown in Fig. 14b. Up to 40 cm, the number and length of fractures in the overlying rock layer increased significantly, resulting in the expansion of the “trapezoidal” structure formed by the fractures. The presence of vertical fissures in the overburden cleavage makes the overburden cleavage network more complex, as shown in Fig. 14c. When advancing to 50 cm, the fissure development of the overlying rock layer is more active, and the fissure develops upward through the overlying rock layer on the working face of the upper disc of the No. 5 coal seam, and the number of small fissures on both sides of the fault that do not affect the structural stability increases. Due to the influence of mining in the upper disc workings of the 5^#^ seam, a small amount of fissures were also generated in the direct top overburden rock of the 5^#^ seam, as shown in Fig. 14d. Compared with the fissure development map of the lower disc, it can be obtained that the overburden fissures in the upper disc of the 5^#^ coal seam are more developed, and the height and range of development are also larger. As a result of the mining influence of the lower disc mining and the effect of faults, the completion of the lower disc mining has caused a significant increase in the stress of the upper disc. It can be seen from the cloud diagram of vertical stress distribution of overburden rock, with the increase of the working face mining distance, the stress on the overburden rock and the rock layer of the bottom plate above the mining hollow area is increasing in a “trapezoidal” structure. After the end of mining back, the stresses are mainly concentrated in the fault and the protective coal pillar, as shown in Fig. 14.

**Fig. 15:**
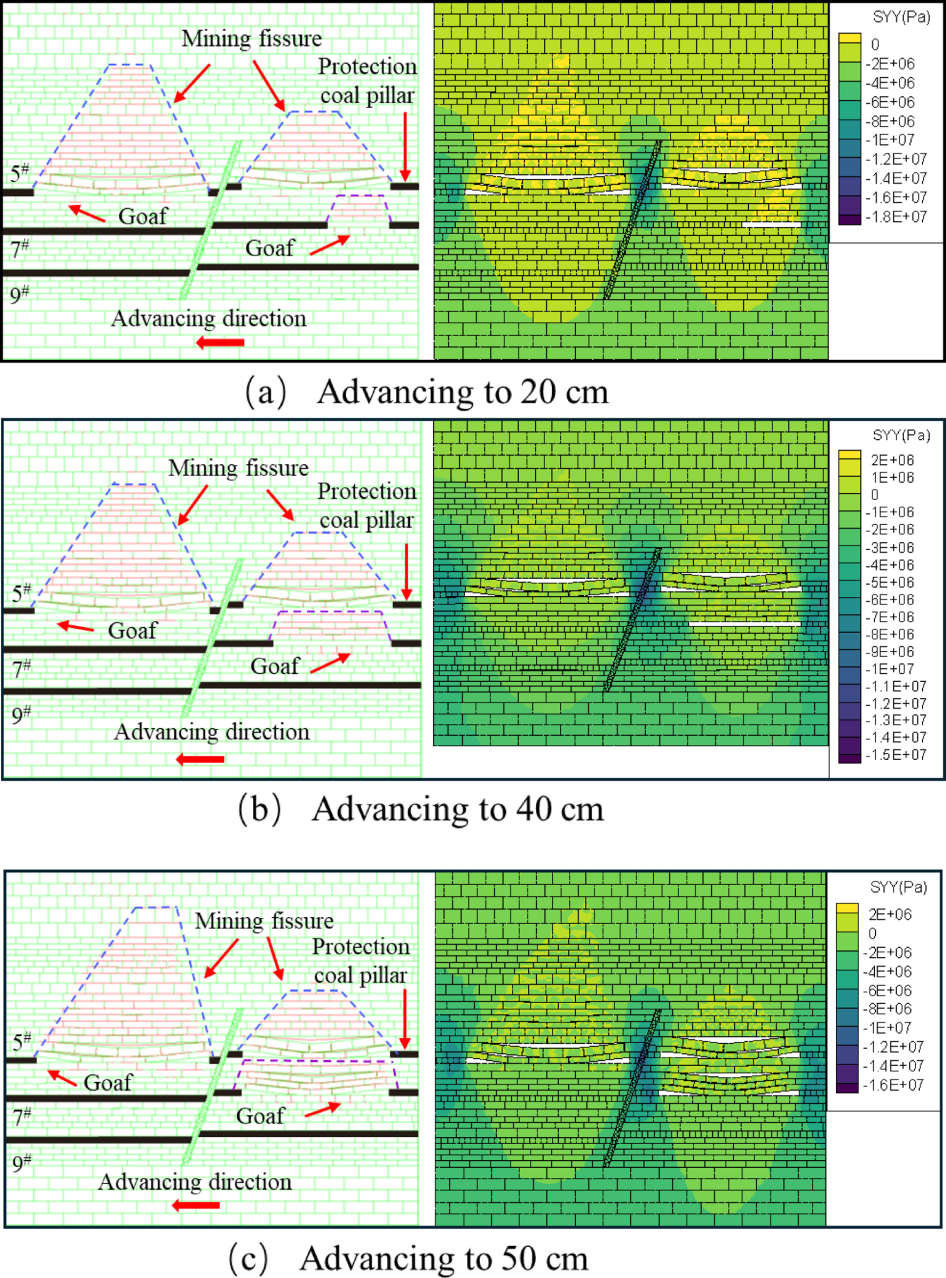
Map of excavation fissure development and stress change in the lower disc of 7^#^ coal seam.

When the lower face of the 7^#^ coal seam advances to 30 cm, the overburden rock above the fissure penetration is in a “trapezoidal” structure, and a small number of horizontal fissures and vertical fissures are generated in the bottom plate, as shown in Fig. 15a. The overburden fissure length increases horizontally and remains constant vertically as the distance mined back increases. The bottom plate produced a small number of horizontal and vertical fractures but did not penetrate to the direct top of the 9^#^ coal seam. As shown in Fig. 15b, c. It is obvious from the fracture development map that the number of fine fractures on the faults increased significantly after the end of the workover. Compared with the side of the lower disc working face, the number of the side of the upper disc working face is more. Observing the stress distribution cloud diagram, we can see that after the end of the face mining, the stress is mainly concentrated on the faults and the protective coal pillars. As the upper working face of the 7^#^ coal seam has not been mined yet, the stress on the lower working face side of the fault is greater than that on the upper working face side, which is also the reason for the unequal number of fissures produced on both sides of the fault.

**Fig. 16:**
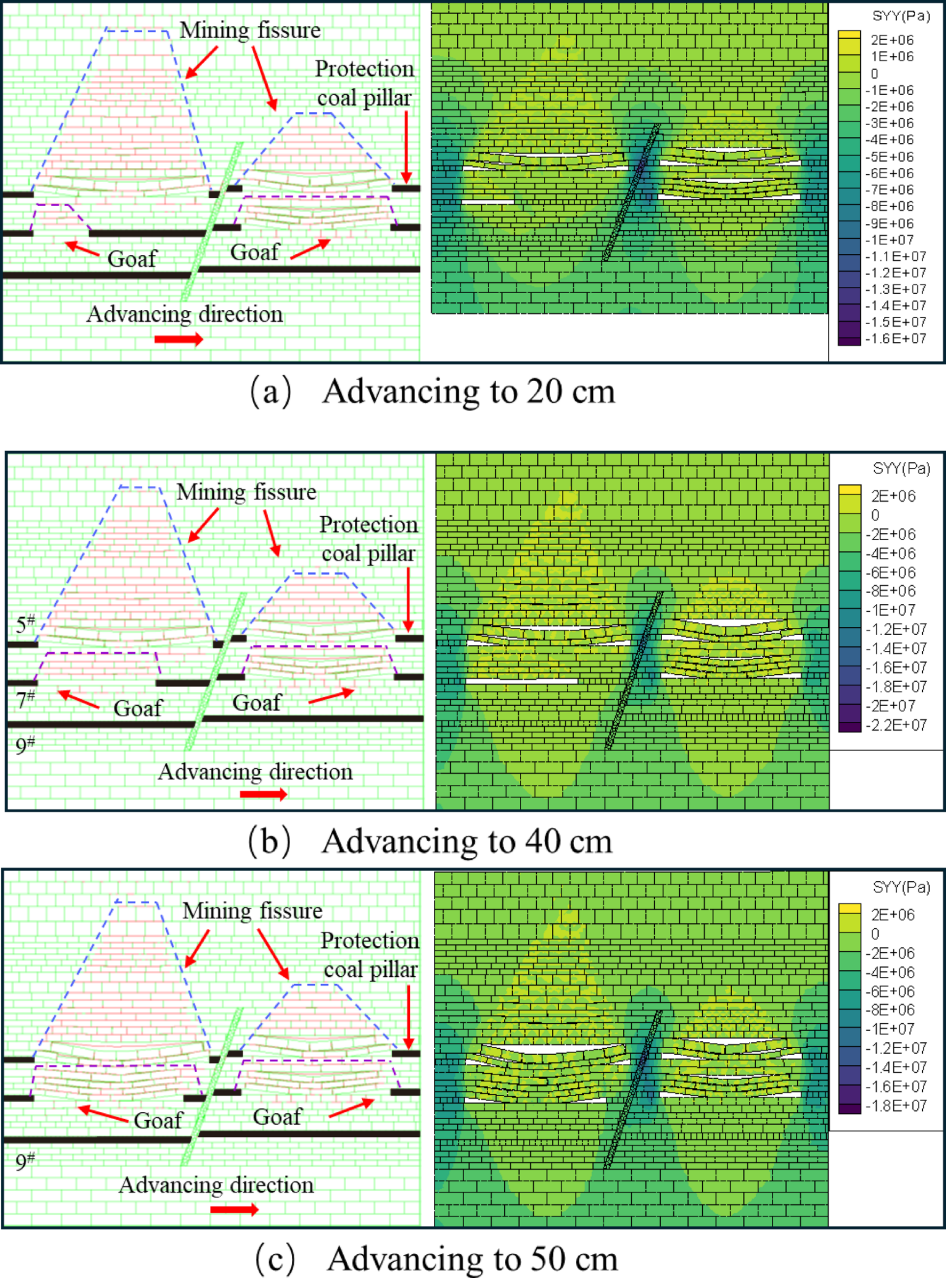
Map of excavated fissure development and stress change in the upper disc of 7^#^ coal seam.

When the upper face of the 7^#^ coal seam advances to 30 cm, the fissures in the overlying rock layer are distributed in a trapezoidal-like structure, and the bottom plate generates a small number of horizontal and vertical fissures through the overlying rock above, as shown in Fig. 16a. When advanced to 40 cm, the overlying rock above the direct top of the unmined area produced a long fissure, but the direct top did not produce a fissure. The fissures in the overlying rock layers in the mining area are further developed the “trapezoidal” network structure is further enlarged, and the direct roof collapse appears to be off-layer fissures, as shown in Fig. 16b. When pushed to 50 cm, the overburden rock above the hollow area lost the support of the coal seam, the overburden rock started to unload, the overburden rock fissure further developed, and a small amount of fissure was generated in the bottom plate at the same time, as shown in Fig. 16c. From the stress distribution cloud diagram, it can be obtained that: in the process of mining back, the top and bottom plate stresses of the upper face of the 7^#^ coal seam are re-altered by the influence of mining, so that the unmined area is subjected to a certain degree of tensile force and pressure and other multi-stress field coupling, forming a class of “trapezoidal” structural distribution. The distribution of the structure is similar to “trapezoidal”.

**Fig. 17:**
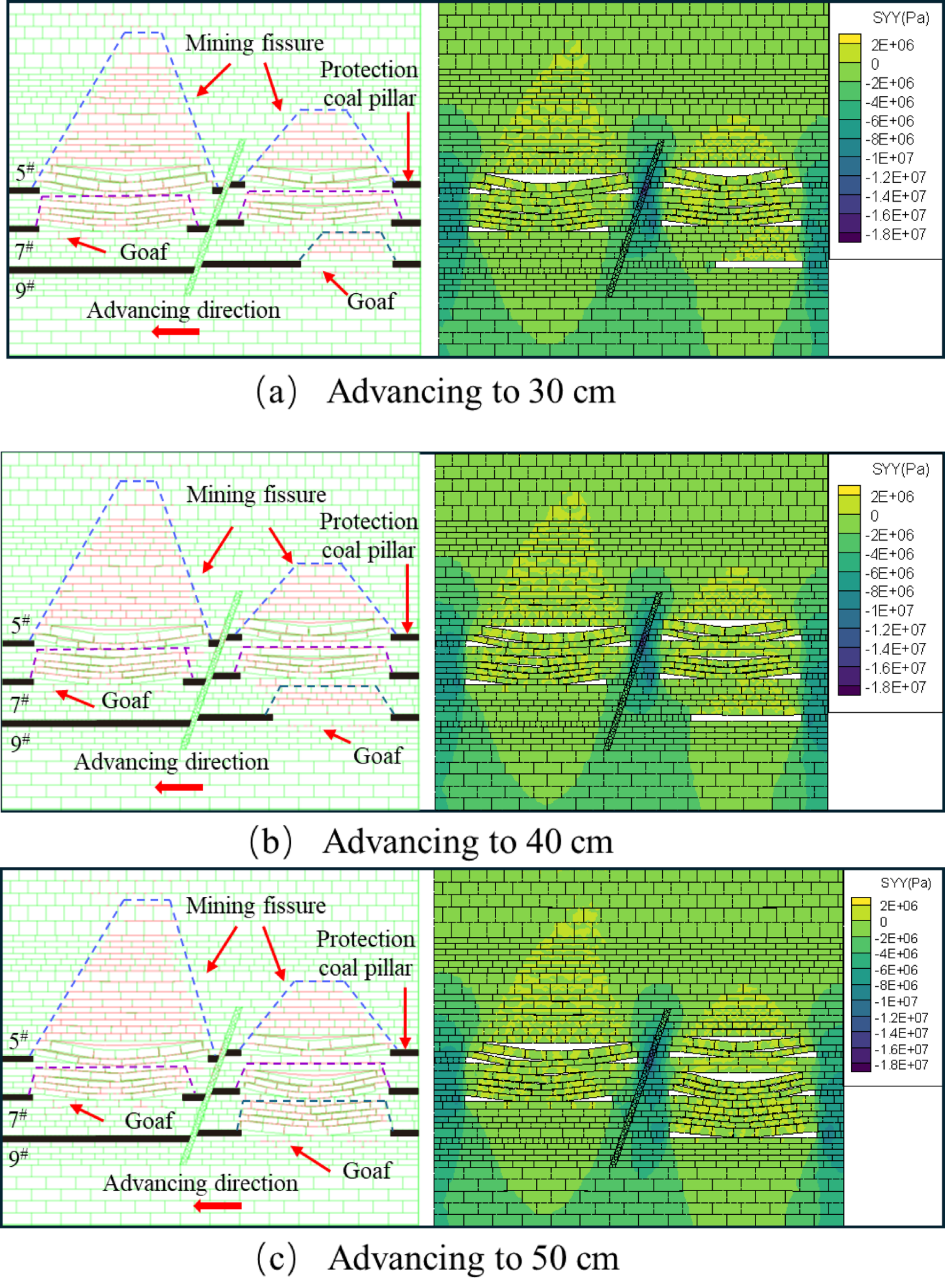
Map of excavated fissure development and stress changes in the lower disc of 9^#^ seam.

**Fig. 18:**
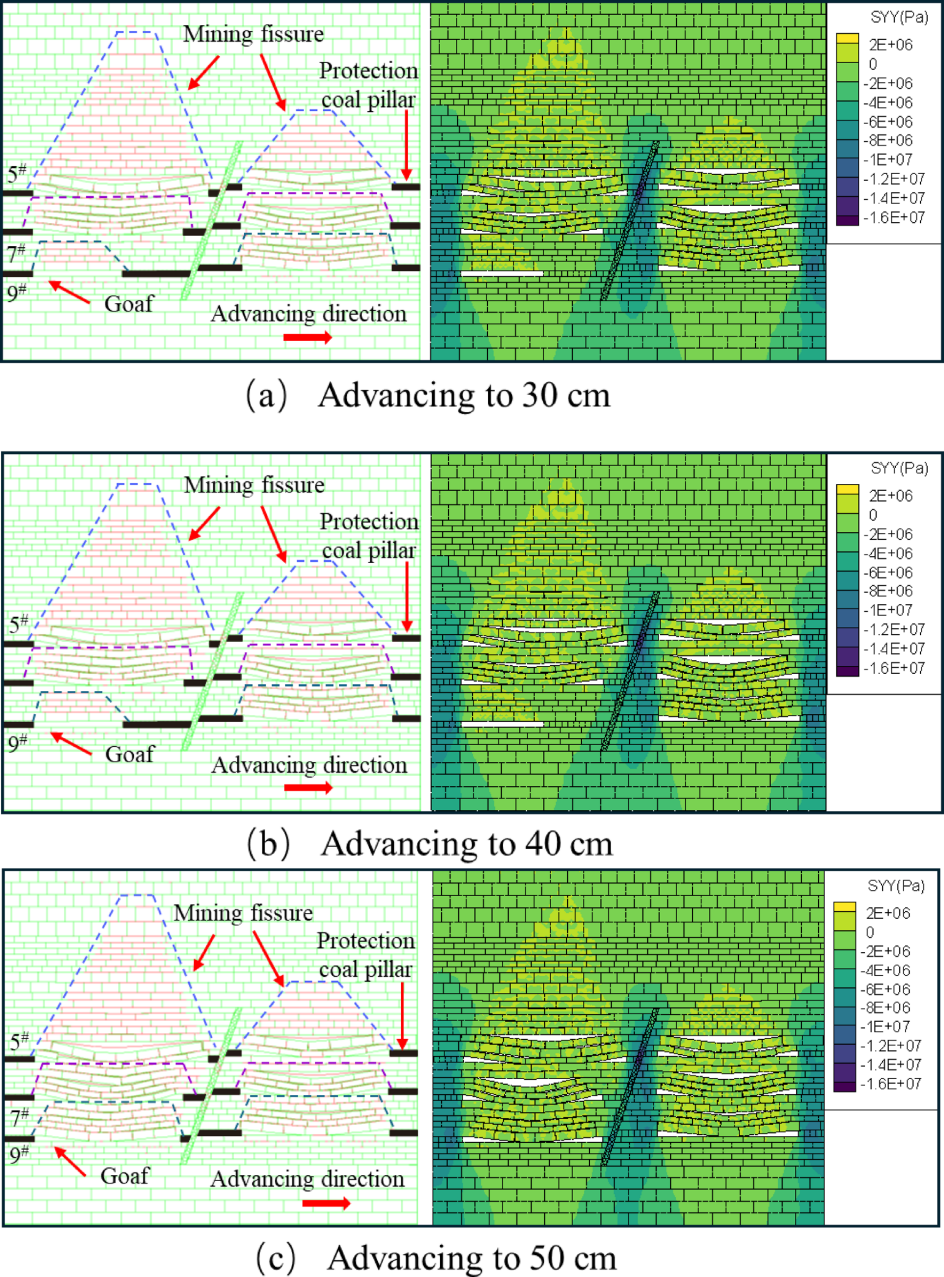
Map of excavated fissure development and stress changes in the upper disc of the 9^#^ seam.

After the end of mining in the lower and upper faces of the 7^#^ seam, the fissures in the overlying rock layer in the mining area are in the form of a “trapezoidal” structure, with the direct roof bending and sinking, and the overlying rock above the direct roof forming a “masonry beam” structure. The fissures of the overlying rock layer on the working face of the 5^#^, 7^#^, and 9^#^ coal seams are penetrated, forming a stable fissure network structure, as shown in Figs. 17 and 18. From the stress distribution map: after the end of the mining work and the collapse and stabilization of the overlying rock layer in the mining area, the stress is concentrated in the central part of the fault, mainly distributed in the opening eye, stop line and fault, and the rock body of the fault zone produces a new stress concentration in the position close to the coal seam, and the largest stress concentration area is located in the central part of the fault.

## Conclusion

This study provides a double verification basis for engineering design by combining visual verification of the physical similarity test with quantitative analysis of numerical simulation. The results of the similar material test and numerical simulation are in good agreement, which jointly reveal the cross-layer linkage mechanism of overburden fracture in group mining, and provide a double verification basis for engineering design.

1. Faults play an important role in the stable structure of coal seams. The experimental results show that the location of the overburden fracture lines caused by mining activities interacts with the faults to create a stable inverted triangular overburden structure pattern when the upper face of the mining disc is close to the fault location. This structure not only helps to strengthen the stability of the fault but also effectively suppresses the “activation” phenomenon that may occur as a result of external disturbances.
2. There are obvious differences in the development of overlying rock fissures during the mining of the upper and lower coal seams. As the upper seam is mined after the lower seam is mined, the stress distribution of the upper seam is affected by the mining of the lower seam, resulting in an increasing trend in the stress level sustained by the upper seam compared to the stress level of the lower seam under the same mining conditions. This has contributed to the process of fissure development in the upper seam becoming more fully developed, with not only an increase in the number and density of fissures but also a more significant depth and breadth of their extension.
3. Faults play a key role in stress distribution in the overlying rock layers. Numerical simulation results show that the stress distribution in the overlying rock strata of the upper coal seam can be significantly affected by mining activities when mining operations are carried out in the lower coal seam. The stress state of the overlying rock strata in the area adjacent to the protected coal pillar changes significantly, and the denser fractured side near the fault tends to be subjected to a relatively lower level of stress compared to the less fractured side.

## Research limitations analysis and future prospects

### Analysis of research limitations

1. Limitations of experimental methods: Although physical similarity simulation can directly reflect the evolution process of overburden fractures, the simulation accuracy of three-dimensional stress state and complex rock mass structure in fault zone under real geological conditions is limited by the size of the experimental platform and the characteristics of similar materials. For example, it is difficult to completely reproduce the heterogeneity and anisotropy of the actual fault zone by using a single ratio of fault zone material (yellow mud: gypsum: lime = 6:3:1) in the experiment.
2. Simplification of model construction: The Mohr-Coulomb elastoplastic constitutive model used in numerical simulation can better describe the elastoplastic deformation characteristics of coal and rock mass, but it ignores the complex mechanical behaviors such as strain softening and dilatancy in the process of rock mass damage evolution. In addition, the model simplifies the coal seam dip angle as horizontal (0°), which is different from the dip angle of 20°~24° in the actual geological conditions, which may lead to the deviation between the simulation results of stress distribution and fracture evolution law and the field measured data.

### Future prospects

1. Optimization and innovation of experimental methods: A three-dimensional physical simulation experiment platform was introduced to construct a three-dimensional model containing fine structures of fault zones (such as the main fault plane, fracture zone, and influence zone). Combined with digital speckle (DIC), microseismic monitoring, and other technologies, the dynamic evolution of the three-dimensional stress field and fracture network was tracked in real-time. At the same time, similar materials that can simulate groundwater seepage are developed, and mining experiments under the coupling of seepage-stress-fracture are carried out to reveal the influence mechanism of multi-field coupling on the stability of overlying strata.
2. Refined construction of numerical model: More complex constitutive models (such as the damage softening model and visco-elastoplastic model) are used to describe the progressive failure process of coal and rock mass, and the true mechanical parameters of fault zone rock mass are obtained based on CT scanning and acoustic testing to improve the fidelity of numerical simulation. In addition, a three-dimensional numerical model considering the real dip angle of coal seam and multi-structure superposition is established to analyze the three-dimensional distribution characteristics of overburden fractures in strike and dip directions.

## Data Availability

All data, models, and code generated or used during the study appear in the published article.
